# Supplementary material for: ProteinShader: illustrative rendering of macromolecules
Source: BMC Struct Biol. 2009 Mar 30;9:19. doi: 10.1186/1472-6807-9-19 (PMC2672931; doi:10.1186/1472-6807-9-19)
Supplement: Additional file 1 — ProteinShader program without source code. This compressed file contains the complete ProteinShader program including associated libraries, but no source code. A README.txt file gives an overview of the ProteinShader distribution, and the index.html file in the help subdirectory has directions on getting started with the program as well as a set of tutorials. [file 1472-6807-9-19-S1.zip › ProteinShader-beta-0_9_4-binary/help/api/org/proteinshader/structure/class-use/Atom.html]

Uses of Class org.proteinshader.structure.Atom (ProteinShader API)


|  |  |  |
| --- | --- | --- |
| **Overview** \| **Package** \| **Class** \| **Use** \| **Tree** \| **Deprecated** \| **Index** \| **Help** | |  |
| PREV   NEXT | **FRAMES**    **NO FRAMES**     **All Classes** |  |


---


## **Uses of Class org.proteinshader.structure.Atom**

| Packages that use Atom | |
| --- | --- |
| **org.proteinshader.graphics.adapter** | Holds the StructureToGraphics class, which is used to manage the use of the drawing classes and OpenGL display lists. |
| **org.proteinshader.gui** | Holds all of the Swing GUI components and their associated listeners, including class Renderer, which is registered as a listener for the GLCanvas object that is used a drawing surface. |
| **org.proteinshader.gui.components.controlpanel** | Holds the JPanels used in the right-side retractable control panel of the GUI. |
| **org.proteinshader.structure** | Holds the classes that store information from a Protein Data Bank file: Structure, Model, Chain, AminoAcid, Heterogen, Water, Atom, Bond, Helix, BetaStrand, Loop, *etc*. |
| **org.proteinshader.structure.visitor** | Holds Visitor classes that know how to traverse the hierarchy of objects held by class Structure. |
| **org.proteinshader.structure.visitor.modifiers** | Holds helper classes for the ModifierVisitor class, which is used to modify Drawable objects (Atoms, Bonds, and Segments). |

Uses of Atom in org.proteinshader.graphics.adapter

| Methods in org.proteinshader.graphics.adapter with parameters of type Atom | |
| --- | --- |
| `void` | `StructureToGraphics.draw(GL gl, Atom atom)`             Draws an Atom as a sphere. |

Uses of Atom in org.proteinshader.gui

| Methods in org.proteinshader.gui that return Atom | |
| --- | --- |
| `Atom[]` | `MediatorImpl.getCurrentAtoms()`             Returns the current Atoms. |
| `Atom[]` | `Mediator.getCurrentAtoms()`             Returns the current Atoms. |

| Methods in org.proteinshader.gui with parameters of type Atom | |
| --- | --- |
| `void` | `MediatorImpl.setCurrentAtoms(Atom[] atoms)`             Sets the current Atoms. |
| `void` | `Mediator.setCurrentAtoms(Atom[] atoms)`             Sets the current Atoms. |

Uses of Atom in org.proteinshader.gui.components.controlpanel

| Methods in org.proteinshader.gui.components.controlpanel that return Atom | |
| --- | --- |
| `Atom` | `SelectorPanel.getSelectedAtom()`             Returns the currently selected Atom from the Atom list. |
| `Atom[]` | `SelectorPanel.getSelectedAtoms()`             Returns an array of the currently selected Atoms from the Atom list. |

Uses of Atom in org.proteinshader.structure

| Methods in org.proteinshader.structure that return Atom | |
| --- | --- |
| `Atom` | `AminoAcid.addNewAtom(int serialNo, AtomEnum type, String atomID, double temperature, int charge, double occupancy, String altLocation, double x, double y, double z, VisibilityEnum visibility)`             Creates a new Atom with the type and Atom ID given as arguments, adds the Atom to the Residue's collection of Atoms, and returns a reference to the new Atom. |
| `Atom` | `Residue.addNewAtom(int serialNo, AtomEnum type, String atomID, double temperature, int charge, double occupancy, String altLocation, double x, double y, double z, VisibilityEnum visibility)`             Creates a new Atom with the type and Atom ID given as arguments, adds the Atom to the Residue's collection of Atoms, and returns a reference to the new Atom. |
| `Atom` | `Structure.addNewAtom(int atomSerialNo, AtomEnum atomType, String atomID, String aminoAcidType, String residueID, String chainID, String modelID, double temperature, int charge, double occupancy, String altLocation, double x, double y, double z, VisibilityEnum visibility)`             Creates a new atom with the atomID given as an argument, adds the new Atom to the requested Model-Chain-AminoAcid, and then returns a reference to the new Atom. |
| `Atom` | `Structure.addNewHetAtom(int atomSerialNo, AtomEnum atomType, String atomID, String hetName, String residueID, String chainID, String modelID, double temperature, int charge, double occupancy, String altLocation, double x, double y, double z, VisibilityEnum visibility)`             Creates a new atom with the atomID given as an argument, adds the new Atom to the requested Model-Chain-(Heterogen or Water), and then returns a reference to the new Atom. |
| `Atom` | `Atom.getAltAtom()`             Returns an alternate Atom with a lower occupancy (if it exists). |
| `Atom` | `Structure.getAtom(int atomSerialNo)`             Returns the Atom with Atom serial number given as an argument (or null if not found). |
| `Atom` | `Residue.getAtom(String atomID)`             Returns the Atom with the atomID given as an argument, or returns null if the Atom is not found. |
| `Atom` | `Structure.getAtom(String atomID, String residueID, String chainID, String modelID)`             Returns the Atom with the IDs given as arguments. |
| `Atom` | `AminoAcid.getC()`             Returns the carbon Atom of the carbonyl group if it exists. |
| `Atom` | `AminoAcid.getCA()`             Returns the alpha carbon Atom if it exists. |
| `Atom` | `Bond.getDstAtom()`             Returns the destination Atom for this Bond. |
| `Atom` | `AminoAcid.getHAor1HA()`             Returns the hydrogen Atom on the alpha carbon if it exists. |
| `Atom` | `AminoAcid.getHorHN()`             Returns the hydrogen Atom on the nitrogen of the amino group. |
| `Atom` | `AminoAcid.getN()`             Returns the nitrogen Atom of the amino group if it exists. |
| `Atom` | `AminoAcid.getO()`             Returns the oxygen Atom of the carbonyl group if it exists. |
| `Atom` | `Water.getO()`             Returns the oxygen Atom (or null if not found). |
| `Atom` | `AminoAcid.getOXT()`             Returns the Chain terminating oxygen if it exists. |
| `Atom` | `Bond.getSrcAtom()`             Returns the source Atom for this Bond. |

| Methods in org.proteinshader.structure that return types with arguments of type Atom | |
| --- | --- |
| `List<Atom>` | `Structure.getAtomSerialNoCache()`             Returns the Atom serial number cache as a list. |
| `Iterator<Atom>` | `Residue.iteratorAtoms()`             Returns an Iterator for the Atoms held by this Residue. |
| `Iterator<Atom>` | `AminoAcid.iteratorBackboneAtoms()`             Returns an Iterator for the backbone Atoms held by this Residue. |
| `Iterator<Atom>` | `AminoAcid.iteratorSideChainAtoms()`             Returns an Iterator for the side chain Atoms held by this Residue. |

| Methods in org.proteinshader.structure with parameters of type Atom | |
| --- | --- |
| `void` | `Atom.addNewBond(Atom dstAtom, BondEnum type, VisibilityEnum visibility)`             Adds a new Bond with this Atom as the source Atom and the Atom given as an argument as the destination Atom. |
| `Bond` | `Atom.getBond(Atom dstAtom)`             Returns the Bond from this Atom (the source) to the destination Atom. |
| `void` | `Atom.removeBond(Atom dstAtom)`             Removes the Bond from this Atom (the source) to the destination Atom. |
| `void` | `Atom.setAltAtom(Atom atom)`             Sets the alternate Atom. |

Constructors in org.proteinshader.structure with parameters of type Atom

`Bond(Atom srcAtom,
Atom dstAtom,
BondEnum type,
VisibilityEnum visibility)`
  
          Constructs a Bond for the source and destination Atoms given as
arguments.

Uses of Atom in org.proteinshader.structure.visitor

| Methods in org.proteinshader.structure.visitor with parameters of type Atom | |
| --- | --- |
| `Quaternion` | `FrenetFrameGeneratorVisitor.calculateRotation(Atom atom1, Atom atom2, Atom atom3)`             Calculates a rotation matrix [N B T] based on the xyz-centers of the three Atoms given as arguments, and then converts the rotation matrix into a Quaternion that is returned. |
| `boolean` | `BondPredictor.isDistanceAcceptable(Atom srcAtom, Atom dstAtom)`             Determines if two Atoms are within an acceptable distance for a covalent Bond. |
| `boolean` | `BondPredictor.isDoubleBond(Atom srcAtom, Atom dstAtom, AminoAcidEnum aminoAcidType)`             Uses the Atom IDs (from Atom name field in a PDB record) and the AminoAcid type to determine if the Atoms should have a double Bond (assuming that they both belong to the same AminoAcid). |
| `void` | `ModifierVisitor.visit(Atom atom)`             If an AtomModifier has been added, it will be used to modify the Atom. |
| `void` | `Visitor.visit(Atom atom)`             Traverses all Bonds owned by the Atom. |
| `void` | `SFWriterVisitor.visit(Atom atom)`             Writes all information held by an Atom. |
| `void` | `BoundsVisitor.visit(Atom atom)`             Checks xyz-coordinates to look for any new min or new max. |
| `void` | `VisibilityVisitor.visit(Atom atom)`             Tests the visiblity of the Atom in order to determine if it should be transferred to the opaque Atoms and Bonds list or the translucent Drawables list. |
| `void` | `BondDestroyerVisitor.visit(Atom atom)`             Removes all Bonds on the Atom. |
| `void` | `BondGeneratorVisitor.visit(Atom atom)`             Checks if the Atom is a possible Bond destination Atom for any previously seen Atoms of the same Residue, and then adds the Atom to the list of Atoms to remember. |

Uses of Atom in org.proteinshader.structure.visitor.modifiers

| Methods in org.proteinshader.structure.visitor.modifiers with parameters of type Atom | |
| --- | --- |
| `void` | `AtomModifier.modify(Atom atom)`             Modifies the ball radius if requested, and then calls on modify() of the superclass, DrawableModifier, to apply any other requested modifications such as visibility, color, or radius for space-filling model. |

---


|  |  |  |
| --- | --- | --- |
| **Overview** \| **Package** \| **Class** \| **Use** \| **Tree** \| **Deprecated** \| **Index** \| **Help** | |  |
| PREV   NEXT | **FRAMES**    **NO FRAMES**     **All Classes** |  |


---

# *Copyright © 2007-2008*
